# Improvement of immune thrombocytopenia with imatinib therapy following chronic myeloid leukemia

**DOI:** 10.1007/s12185-022-03492-9

**Published:** 2022-11-14

**Authors:** Yuichi Nakamura, Yoshihiro Itoh, Naoki Wakimoto

**Affiliations:** Department of Hematology, Saitama Medical University Hospital, 38 Morohongo, Moroyama-Machi, Iruma-Gun, Saitama, 350-0495 Japan

**Keywords:** Immune thrombocytopenia, Chronic myeloid leukemia, Tyrosine kinase inhibitor, Imatinib, Immunological off-target effects

## Abstract

Immune thrombocytopenia (ITP) and chronic myeloid leukemia (CML) are rarely observed concurrently. Here we report the case of a patient with ITP who developed CML that has been well controlled with tyrosine kinase inhibitor (TKI) therapy. A 55-year-old man was diagnosed with ITP. No cytogenetic abnormalities were found at the time of initial diagnosis. Four years later, he began corticosteroid therapy for progression of thrombocytopenia. At that time, the Philadelphia (Ph) chromosome was observed in 7 of 20 bone marrow (BM) cells, suggesting concurrent CML in the subclinical stage. Prednisolone resulted in a partial response. Seven months after starting prednisolone, he exhibited hematological features of CML with an increase in Ph-positive cells. TKI therapy with imatinib mesylate was started to treat CML and maintained at a daily dose of 400 mg. The patient achieved and sustained a major molecular response. His platelet count also increased, enabling discontinuation of corticosteroid therapy. TKIs have been reported to show various immunological off-target effects. In this case, off-target effects of TKI might have improved ITP by suppressing the autoimmune response. Alternatively, reconstitution of immune systems by Ph-negative cells or cancellation of immunoreaction against CML could have exerted favorable effects on ITP.

## Introduction

Immune thrombocytopenia (ITP) is an autoimmune disease characterized by anti-platelet autoantibodies, due to an abnormal T cell response that stimulates the proliferation and differentiation of autoreactive B cells [1]. Although the association of autoimmune disorders and hematologic neoplasms has been well recognized, ITP and chronic myeloid leukemia (CML) are rarely observed concurrently [2–6]. Here we report a patient with ITP who developed CML during the course of the disease. Corticosteroid therapy for ITP resulted in only a partial response, but thrombocytopenia was improved after the subsequent tyrosine kinase inhibitor (TKI) therapy for CML, suggesting TKI would have exert favorable effects on ITP.

## Case report

A 55-year-old male was referred to our hospital because of thrombocytopenia. He has history of hypertension, hyperuricemia, diabetes mellitus (DM) and hyperlipidemia. DM had required insulin therapy. He did not present any abnormal bleeding. Liver or spleen was not palpable on physical examination of abdomen. Peripheral blood showed hemoglobin 144 g/L, platelets 29 × 10^9^/L, and white blood cells (WBC) 4.8 × 10^9^/L with 75% neutrophils, 3% eosinophils, 6% monocytes, and 16% lymphocytes (Table 1). Blood coagulation tests were normal. Bone marrow (BM) examination presented normocellular marrow with slight increase of megakaryocytes. Infiltration of abnormal cells into BM or any dysplastic changes of hematopoietic cells were not observed. G-banded chromosomal analysis of BM cells presented normal karyotype. Platelet-associated immunoglobulin G (PAIgG) value was elevated to 161 ng/10^7^ cells. Serum IgG antibody against *Helicobacter pylori* was negative. Anti-nuclear antibody test was positive with a titer of 1:80 and homogenous and speckled patterns. Anti-cardiolipin antibody, anti-dsDNA antibody or anti-Ro/SSA antibody was negative. He did not fulfill the diagnostic criteria of systemic lupus erythematosus or antiphospholipid syndrome. He was diagnosed with ITP, but had not received therapy except for intravenous immunoglobulin at the time of endoscopic resection of colonic adenomas, keeping platelet count of 20 to 30 × 10^9^/L.

**Table 1:** Peripheral blood, bone marrow, and cytogenetic findings during the course of diseases

	At the diagnosis of ITP	At the start of prednisolone	At the start of imatinib
WBC (× 10^9^/L)	4.8	6.2	9.6
Myelocytes (%)	0	1	3
Metamyelocytes (%)	0	0	1
Band and segmented forms (%)	75	66	64
Eosinophils (%)	0	4	1
Basophils (%)	0	1	3
Monocytes (%)	6	5	5
Lymphocytes (%)	16	23	23
Hemoglobin (g/L)	144	152	152
Platelet (× 10^9^/L)	29	7	25
Bone marrow			
Cellularity	Normocellular	Normocellular	Slightly hypercellular
Number of megakaryocytes	Slight increase	Increase	Increase
Blasts (%)	2.0	1.9	1.2
Granulocytes (%)	46.8	52.1	71.4
Erythroblasts (%)	19.8	30.5	20.5
Ph-positive cells	0/20	7/20	14/20

Cellularity and number of megakaryocytes in bone marrow were evaluated on the basis of histological findings of clot section of aspirated samples

Four years after the first visit to our hospital, he developed epistaxis with decrease of platelet count to 10 × 10^9^/L and was admitted for corticosteroid therapy for ITP. On admission, peripheral blood showed hemoglobin 152 g/L, platelets 7 × 10^9^/L, and WBC 6.2 × 10^9^/L without definite abnormalities in hemogram (Table 1). Unexpectedly, G-banded chromosome analysis of BM cells showed 46,XY,t(9;22)(q34;q11)[7]/46,XY[13], indicating concurrent chronic phase of CML in subclinical stage. *BCR*-*ABL*1 chimeric transcript corresponding to P210 b2a2 type was confirmed by reverse transcriptase polymerase chain reaction analysis.

**Fig. 1:**
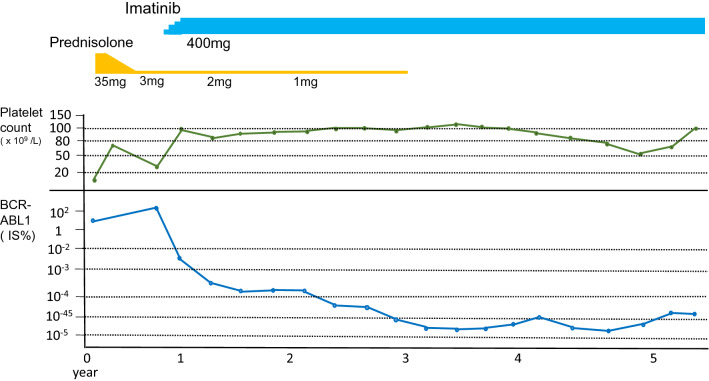
Clinical course of the patient. His treatment course, platelet count, value of platelet-associated immunoglobulin G, and amount of BCR-ABL1 transcript are shown. BCR-ABL1 was measured by quantitative RT-PCR from peripheral cells and evaluated by IS. *IS* International Scale

For ITP, prednisolone was started at a daily dose of 35 mg (0.5 mg/kg), resulting in a partial response in which platelets increased to 77 × 10^9^/L at maximum. Because of the worsening of DM, prednisolone was reduced in dose and maintained at 3 mg daily (Fig. 1). Seven months after the start of prednisolone, he presented hemoglobin 152 g/L, platelets 25 × 10^9^/L, and WBC 9.6 × 10^9^/L with immature neutrophils and basophils in hemogram recognized as feature of chronic phase of CML (Table 1). BM showed myeloid hyperplasia without increase of blasts and chromosome analysis showed 46,XY,t(9;22)(q34;q11)[14]/46,XY[6]. Partial improvement of thrombocytopenia and hematological manifestation with the increase of Ph-positive cells led us to start TKI therapy for CML.

Imatinib mesylate was started and maintained at a daily dose of 400 mg. TKI of the second generation, nilotinib or dasatinib was not adopted because of coexistence insulin-dependent DM or risk of hemorrhagic complications.

At 6 months, he achieved cytogenetic and major molecular response. Simultaneously, platelet count was elevated up to 116 × 10^9^/L. Prednisolone was further reduced in dose and was finally stopped 2 years after the start of imatinib. As to CML, he reached to molecular remission (MR^4.0^) [7] at 16 months, which has been maintained for 3 years until now. Platelet count has also been sustained at 50 to 100 × 10^9^/L without corticosteroid (Fig. 1).

## Discussion

Here we present a case with ITP who developed CML during the course of the disease. Cases with CML after diagnosis of ITP were rarely reported. However, the nationwide large-scale investigation on Swedish population revealed that the prevalence of prior autoimmune disease as well as malignancies was elevated in CML patients compared with matched controls [6]. In this study, two cases with ITP diagnosed before CML were listed, although their clinical features were not described. In the literature, four case reports of CML after diagnosis of ITP were found (Table 2). Two of them were pediatric ITP cases who later became refractory to corticosteroids and splenectomy and developed CML 7.5 or 29 years after the initial diagnosis of ITP [2, 3]. The others were adult cases who developed CML 5 or 15 years after the diagnosis of ITP [4, 5]. In these case reports, at the diagnosis of CML, platelet count remained low in one case, normalized in another case, and elevated in the other two cases. Notably, both of adult cases had been treated by eltrombopag, a thrombopoietin (TPO) receptor agonist, after ITP became refractory to corticosteroids [4, 5]. In these reports, the possibility was postulated that long-term use of TPO receptor agonist would promote the onset of CML.

**Table 2:** Previous and current cases of CML after diagnosis of ITP

Case	References	Gender	Age at the diagnosis of ITP (year)	Platelet count at the diagnosis of ITP (× 10^9^/L)	Therapy for ITP	Age at the diagnosis of CML (year)	Platelet count at the diagnosis of CML (× 10^9^/L)	Clinical outcome of CML
1	[2]	F	8	30	Corticosteroids, splenectomy, 6-MP	15	168	Died without blastic crisis after busulfan
2	[3]	M	3	5	Corticosteroids, splenectomy, IVIG	32	> 100	Good response by imatinib
3	[4]	M	72	NA	Corticosteroid, eltrombopag	77	50	Died in blastic crisis after imatinib and dasatinib
4	[5]	M	49	NA	Corticosteroid, eltrombopag	64	800	Good response by imatinib
5	Present case	M	55	29	IVIG, corticosteroid	60	7	Good response by imatinib

Additional two cases were reported, but their clinical information was not available [6]

*CML* chronic myelogenous leukemia, *ITP* immune thrombocytopenia, *6-MP* 6-mercaptopurine, *IVIG* intravenous immunoglobulin, *NA* not assessed

The present case was diagnosed as CML at early stage of disease without manifestation of leukocytosis. Corticosteroid therapy for ITP resulted in only a limited response. After the start of TKI therapy, persistent thrombocytopenia was improved, enabling discontinuation of corticosteroid therapy.

If TKI was also effective for ITP, one possible mechanism would be the immunosuppression by its off-target effects. TKIs have various immunological off-target effects, modulating protein tyrosine kinases involved in key signaling pathways in both effector and regulatory immune cells [8]. For instance, imatinib has been shown to inhibit the differentiation of dendritic cells and induction of primary cytotoxic T-lymphocyte response mediated via reduced phosphorylation of AKT/PKB and nuclear accumulation of NF-κB [9, 10]. Another study has presented the impairment of FLT3L-mediated dendritic cell expansion by imatinib [11]. TKIs have also been shown to induce the impairment of B cell signaling and survival through inhibition of Bruton’s tyrosine kinase phosphorylation, leading to the reduction in the numbers of IgM-producing memory B cells and the humoral responses to influenza and pneumococcal vaccination [12]. Furthermore, studies have reported that imatinib inhibits T cell proliferation through the inhibition of LCK and ERK1/2 phosphorylation and NF-κB activation [13] and induces T cell lymphopenia through the inhibition of STAT5 phosphorylation in response to IL-7 signaling [14]. In our case, these immunosuppressive effects of TKI might have suppressed autoimmune response, leading to the improvement of ITP.

The previous study revealed that Ph chromosomes were found in myeloid cells and in most B cells, but not in mature T cells or natural killer cells in CML, although multipotent hematopoietic stem cells were affected in the disease [15]. In the present case, ITP preceded the onset of CML and subsequently occurring coexistence of ITP and CML suggested that autoreactive B cells survived even after hematopoietic and immune systems were substituted by the CML clone. It was not determined whether CML cells had enhanced pathological immunoreaction by BCR-ABL1 tyrosine kinase or not. However, the possibility would be postulated that reconstitution of immune systems after TKI therapy had exerted favorable effects on ITP as an alternative explanation for disease improvement. Additionally, if anti-tumor immunoreaction against CML cells induced or enhanced anti-platelet autoimmunity, cancellation of that with TKI therapy would be another reason for improvement of ITP.

Coexistence of CML and autoimmune disease may be a rare, but recurrent situation. Further investigation is needed for treatment of patients with such complications and accumulation of such rare cases could be useful for developing novel treatments for refractory ITP.

## Data Availability

Data sharing is not applicable to this article as no new data were created or analyzed in this study.
